# Loss of tropical moist broadleaf forest has turned Africa’s forests from a carbon sink into a source

**DOI:** 10.1038/s41598-025-27462-3

**Published:** 2025-11-28

**Authors:** Pedro Rodríguez-Veiga, Joao M. B. Carreiras, Shaun Quegan, Janne Heiskanen, Petri Pellikka, Hari Adhikari, Arnan Araza, Martin Herold, Oliver Cartus, Thomas Luke Smallman, Mathew Williams, Chukwuebuka J. Nwobi, Narumasa Tsutsumida, Casey M. Ryan, Thom Brade, Nezha Acil, Heiko Balzter

**Affiliations:** 1 Institute for Environmental Futures, University of Leicester, University Road, Leicester, LE1 7RH UK; 2 National Centre for Earth Observation, Space Park Leicester, 92 Corporation Road, Leicester, LE4 5SP UK; 3 Present Address: Sylvera Ltd, London, UK; 4 School of Mathematical and Physical Sciences, University of Sheffield, Sheffield, UK; 5 National Centre for Earth Observation, University of Sheffield, Sheffield, UK; 6 Department of Geosciences and Geography, University of Helsinki, P.O. Box 64, 00014 Helsinki, Finland; 7 Finnish Meteorological Institute, Helsinki, Finland; 8 Finnish Southern Africa Cooperation Institute (FSAI), Schwabe Street 10, Windhoek, Namibia; 9 State Key Laboratory for Information Engineering in Surveying, Mapping and Remote Sensing, Wuhan University, Wuhan, 430079 China; 10 Forest Nepal, Amar Marg 88, C3534, Butwal, 32907 Nepal; 11 Laboratory of Earth Systems and Global Change, Wageningen University and Research, Wageningen, The Netherlands; 12 GFZ Helmholtz Centre for Geosciences, Potsdam, Germany; 13 Gamma Remote Sensing, Gümligen, Switzerland; 14 School of GeoSciences, University of Edinburgh, Edinburgh, UK; 15 National Centre for Earth Observation, University of Edinburgh, Edinburgh, UK; 16 Present Address: West Africa Blue, Port Louis, Mauritius; 17 Graduate School of Science and Engineering, Saitama University, Saitama, Japan

**Keywords:** Biogeochemistry, Climate sciences, Environmental sciences

## Abstract

Africa’s forests and woody savannas have historically acted as a carbon sink, removing atmospheric carbon and storing it as biomass. However, our novel analysis reveals a critical transition from a carbon sink to a carbon source between 2010 and 2017. Using new high-resolution satellite-derived biomass maps, validated with field plots and machine learning techniques, we quantified the aboveground biomass stocks across African biomes over a decade. Between 2007 and 2010, the continent gained 439 ± 66 Tg yr^⁻1^ of aboveground biomass, but from 2010 to 2015 biomass declined by − 132 ± 20 Tg yr^-1^ and from 2015 to 2017 this decline continued with a loss of − 41 ± 6 Tg yr^-1^, primarily driven by deforestation in tropical moist broadleaf forests. Gains in savanna biomass partially offset these losses, likely due to shrub encroachment. Our findings underline the urgent need for implementing policies to halt global deforestation as required by the Glasgow Leaders Declaration to close the global emissions gap. The current ongoing revisions of Nationally Determined Contributions to the Paris Agreement need to be even more ambitious to compensate for the ongoing loss of natural carbon sinks.

**Supplementary Information:**

The online version contains supplementary material available at 10.1038/s41598-025-27462-3.

## Introduction

Africa’s ecosystems play a pivotal role in the global carbon cycle, contributing approximately 20% of global carbon removals through terrestrial net primary production, 40% of the world’s carbon emissions from biomass burning and 20% of emissions from deforestation and forest degradation^1^. Carbon removals occur in forests and woody savannas through photosynthesis, while carbon emissions are largely driven by fire during forest cover loss, shifting cultivation, agricultural burning and fuelwood burning (~0.4 Pg C y^-1^ for Africa)^2^. Biomass burning in savannas mainly burns perennial herbaceous plant matter, which does not add significantly to net carbon emissions but is thought to accelerate the carbon cycle by reducing carbon turnover times^3^.

Despite their critical importance, Africa’s forests and savannas face increasing pressures from anthropogenic and natural disturbances, leading to a decline in their carbon sequestration potential. Understanding these dynamics is essential for addressing the goals of the Paris Agreement and devising effective climate mitigation strategies.

While previous studies have mapped African biomass and carbon stocks, they are limited by coarse spatial resolutions, temporal gaps, or methodological inconsistencies. Notably, the sparse availability of field-based data has hampered precise assessments of biomass changes at fine spatial scales. Recent advancements in satellite remote sensing, such as the GEDI LiDAR and ALOS PALSAR radar systems, combined with more powerful machine learning models, offer an unprecedented opportunity to bridge these knowledge gaps.

**Fig. 1:**
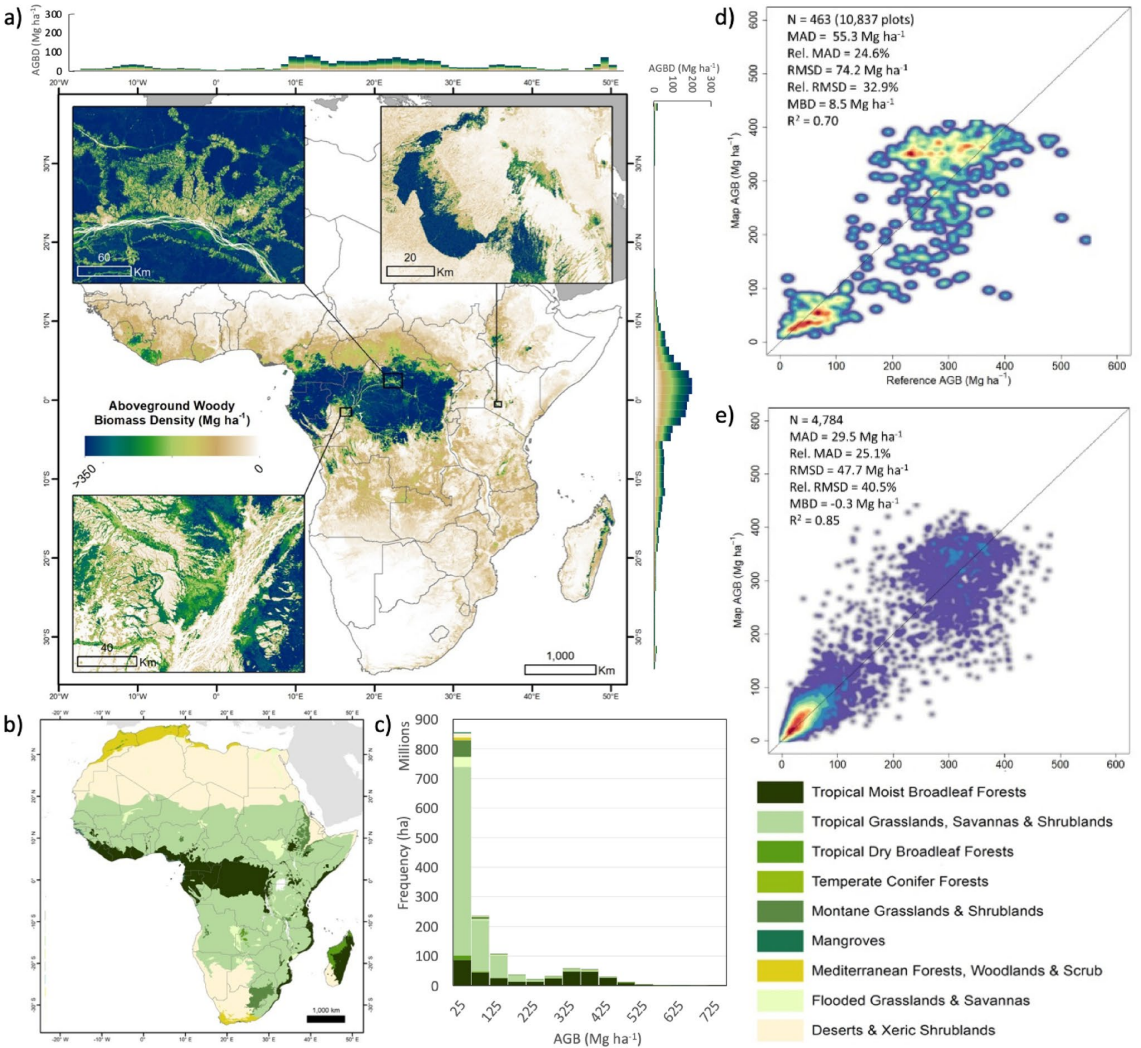
(**a**) Map of African Aboveground Woody Biomass Density (AGBD in Mg ha^-1^) generated at 100 m spatial resolution for the year 2017. Country boundaries were obtained from the geoBoundaries dataset^12^. The map was designed in ArcMap from the ESRI ArcGIS Desktop Suite 10.8.2 (https://desktop.arcgis.com/en)^13^. Histograms along the axes show AGBD averaged over 1 degree longitude and latitude, respectively. Insets show the Mau Forest in Kenya (upper-right), North-East Mongala in DRC (upper-left), and Gamboma in Republic of Congo (lower-left). (**b**) Map of the Terrestrial Ecoregions of the World for Africa^14^ used as biome stratification (designed in ArcGIS). (**c**) Histogram of Aboveground Woody Biomass (AGB in Mg) summed over all biomes. (**d**) Scatterplot of 2017 AGBD map averaged within 0.1⁰ grid cells against a harmonised independent plot AGBD dataset as in^15^. (**e**) Scatterplot of the 2015 and 2016 AGBD against independent airborne LiDAR-based estimates of AGBD, averaged over 300 m grid cells. Warmer colours indicate higher point density, and the black solid line corresponds to the 1:1 line (i.e. perfect agreement).

Previous studies have estimated the extent of African forests, including mosaics of forests and woodlands, as between 638.2 and 836.8 million ha^4^. These forests contain large carbon stocks in the form of aboveground woody biomass, in the range from 85 to 129 Pg^5–9^. African tropical forests have an average aboveground biomass density over 396 Mg ha^-1^, but this can be as high as 429 Mg ha^-1^ in some areas of the Congo Basin^10^. This far exceeds the carbon stock density in savannas with an average aboveground biomass density of 45 Mg ha^-1^^11^, although savannas cover a much larger total area^4^, making forests and savannas the most relevant biomes for understanding the total carbon stocks stored in aboveground woody biomass (Fig. 1).

The quantitative nature of carbon dynamics of African ecosystems remains subject to scientific debate due to the sparse observation network^1,3^. Bombelli et al.^3^ estimated that sub-Saharan Africa is a net carbon sink of between 0.16 - 1 Pg C yr^-1^, depending on the data source used. Bombelli et al.^3^ also concluded that biogeochemical process models give an “unrealistically large sink” between 1.3 and 3.9 Pg C yr^-1^ (average of 3.23 Pg C yr^-1^). Even the direction of the carbon fluxes in Africa is controversial. Some recent studies indicate that the African carbon sink is declining^16^, while other studies conclude that it is in a near-zero steady-state^2^, or even that it has already transitioned to a carbon source^17,18^. A more recent assessment of the African greenhouse gas budget in REgional Carbon Cycle Assessment and Processes 1 (RECCAP1)^19^ concluded that Africa’s carbon sink capacity is decreasing and net ecosystem exchange switched from a small sink of -0.61 ± 0.58 PgC yr^-1^ to a small source in RECCAP2 at +0.16 (CI=[-0.52;1.36]) PgC yr^-1^. In this context, the role of forest degradation^20^, i.e. the partial loss of aboveground tree biomass from a forest, and total forest cover loss, as well as their interannual variability are major uncertainties in the African carbon cycle^1^.

The rationale of this study was to analyse new satellite-based maps of aboveground biomass stocks at unprecedented high spatial resolution and to use the biomass dynamics inferred from these maps to test whether forest biomass stocks in Africa have switched from a net sink into a net source.

Earth observation from satellites combined with field-plot-based national forest inventories provides spatially explicit estimates of key terrestrial carbon pools and their changes over time. In Africa, field plots are scarce. Because of the correlation between forest canopy height, aboveground woody biomass and aboveground carbon stock, satellite-derived forest canopy height maps are the best option for large-scale mapping. Spaceborne Light Detection and Ranging (LiDAR) transmits polarised light from a pulsed laser to measure the distance to the Earth’s surface. Spaceborne LiDAR missions such as ICESAT^21^ and GEDI^22^ allow the estimation of canopy height from the vertical profile of returned laser light to the sensor. These datasets can be used to train empirical and machine learning models, in combination with other datasets such as optical multispectral imagery, digital elevation models, or maps of radar backscatter, in order to map aboveground biomass density across the tropics, including tropical Africa, e.g. for a single year^5,6^ and for different years to estimate biomass (and carbon) gains and losses using coarse spatial resolution images^9,17,23–25^. However, the resolution of these aboveground biomass density maps is too coarse to relate them to drivers of disturbances, such as forest cover loss and forest degradation^26^.

Because deforestation and forest degradation occur on relatively fine spatial scales, more reliable estimates of aboveground biomass density across Africa and its annual gains and losses can only be quantified at high spatial resolution. This is the route to improving our quantitative knowledge of the African terrestrial carbon cycle and estimating carbon emissions and removals more reliably.

## Results

Our analysis of aboveground woody biomass density derived from Earth observation over a time period of 11 years reveals the spatial distribution and temporal trends of biomass gains and losses for all forests and woody savannas in Africa. Fig. 1a shows a map of aboveground woody biomass density for the year 2017. Tropical Grasslands, Savannas and Shrublands are by far the largest biome by area with an average aboveground biomass density of 32 Mg ha^-1^, while Tropical Moist Broadleaf Forests cover a much smaller area with much higher biomass density (Fig. 1b,c). An accuracy assessment against an extensive set of field plot measurements gave a coefficient of determination (R^2^) of 0.70, a Root Mean Square Difference (RMSD) of 74.2 Mg ha^-1^, a Relative Root Mean Square Difference (Rel. RMSD) of 33%, and a Mean Bias Difference (MBD) of 8 Mg ha^-1^, but the higher aboveground biomass densities tended to be overestimated (Fig. 1d). A further comparison with airborne LiDAR-based biomass estimates for 2015 and 2016 (Fig. 1e) showed that the aboveground woody biomass map is unbiased even at higher biomass levels (R^2^ = 0.85, RMSD = 47.7 Mg ha^-1^, Rel. RMSD = 48%, MBD = 0 Mg ha^-1^), though these results may be overoptimistic, due to the spatial proximity to the training data. While both validation approaches suggest there may be potential underestimations beyond 350 Mg/ha (Table S4a), this is a common issue in remote sensing derived AGBD maps. Quantifying AGBD in dense forests is still challenging and AGBD remote sensing retrieval algorithms suffer from large uncertainties at high AGBD^27^.

### Aboveground woody biomass gains and losses across Africa

**Fig. 2:**
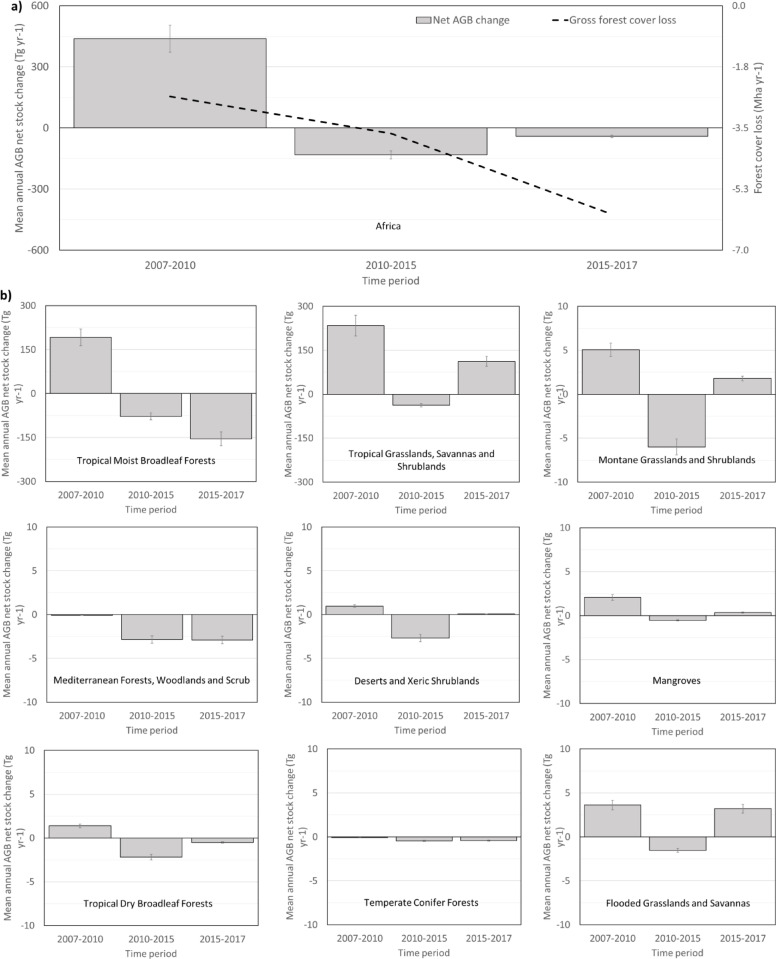

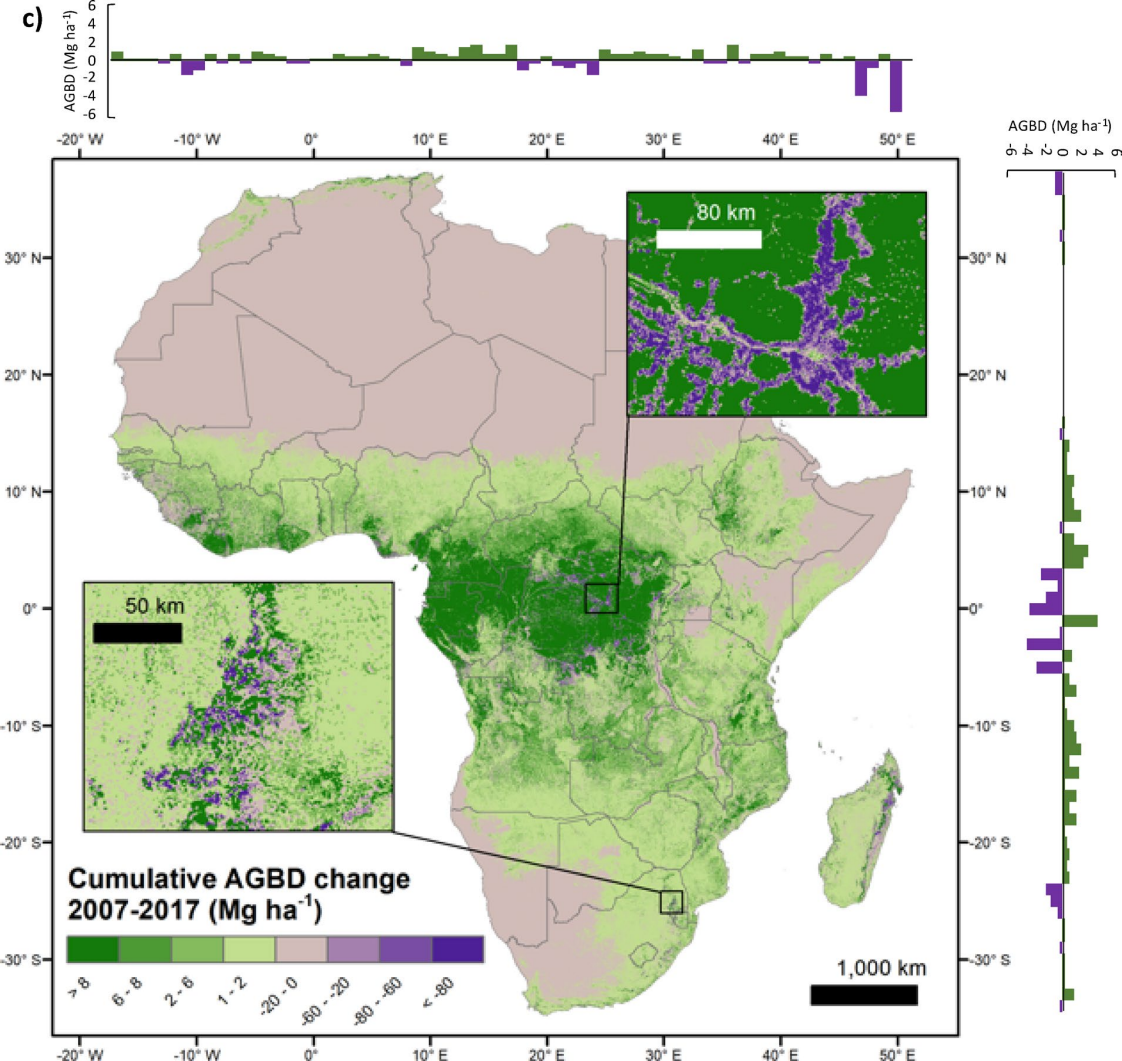
(**a**) Average estimated annual aboveground biomass (AGB) net changes (Tg yr^-1^) for continental Africa for 2007 to 2017 (grey bars). The bars show the average year-to-year change in aboveground biomass. Spaceborne L-band radar data are not available between 2011 and 2014, and so the net annual AGB stock change could not be calculated for that time period. (**b**) The annual gross loss of forest annual net Aboveground Biomass (AGB) change (Tg yr^-1^) per time period and biome. Note that Tropical Moist Broadleaf Forests and Tropical Grasslands, Savannas and Shrublands have a much larger scale on the $y$- axis. Error bars indicate the 95% confidence intervals. (**c**) The map shows cumulative Aboveground Biomass Density (AGBD) net gains (green) and losses (purple) from 2007 to 2017. The map is aggregated to 300 m pixel size for visualization purposes. Only AGB maps from 2007 to 2010 and from 2015 to 2017 were used to generate this map due to the lack of L-band SAR imagery from 2011 to 2014 (See Methods section). The upper right inset shows biomass loss due to forest cover loss occurring around settlements, rivers and roads in Democratic Republic of Congo (DRC), while the lower-left inset is a forest plantation area in South Africa showing clearcuts and newly planted forest. The histograms on the horizontal and vertical axes show AGBD net changes averaged by 1 degree of longitude and latitude, respectively.

From 2007 to 2010, Africa gained 439 ± 66 Tg yr^-1^ (0.37% yr^-1^) net aboveground woody biomass. However, between 2010 and 2015 the continent lost -132 ± 20 Tg yr^-1^ or -0.11% yr^-1^ biomass. Between 2015 and 2017 the average net aboveground biomass change decreased to a loss rate of -41 ± 6 Tg yr^-1^ or -0.03% yr^-1^ (Fig. 2a). These results indicate that Africa’s forests have transitioned from a net sink of aboveground biomass to a net source over that time period. We now investigate where that switch has taken place.

The recent aboveground biomass losses in Africa are mostly driven by losses in the Tropical Moist Broadleaf Forests biome over this period. This is partly offset by biomass gains in the savanna biome from 2015 to 2017 (Fig. 2b). These gains are plausibly explained by enhanced shrub encroachment due to the carbon fertilisation effect of increasing atmospheric carbon dioxide, which has been shown to alter the competition between trees and grasses in savannas in favour of trees^28^. The Tropical Moist Broadleaf Forests biome gained +192 Tg yr^-1^ aboveground biomass in the period 2007 to 2010. This changed to a biomass loss of -70 Tg yr^-1^ between 2010–2015, and an even greater biomass loss of − 154 Tg yr^-1^ between 2015 and 2017. The observed biomass losses for 2007 to 2017 can be explained by a significant increase in forest cover loss rates during the last decade in Democratic Republic of Congo, Madagascar and some West African countries^29^.

**Fig. 3:**
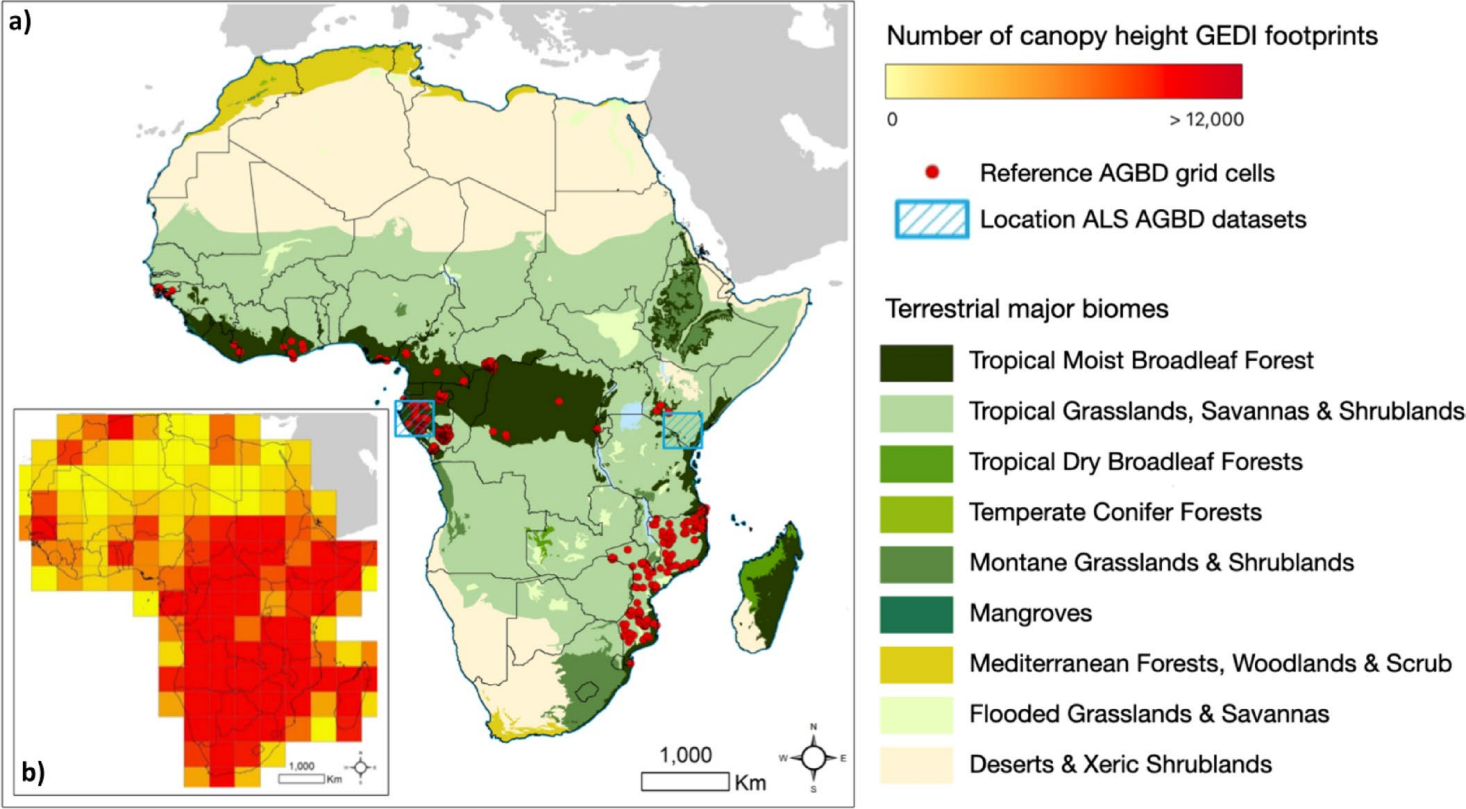
(**a**) Spatial distribution of the 463 0.1° reference grid cells (red dots) derived from the 10,837 field plots used in this study. The map also shows in blue shaded boxes the location of the Airborne Laser Scanning (ALS) datasets used. The background map shows the major terrestrial biomes according to Dinerstein et al.^14^. (**b**) The inlet map shows the number of GEDI footprints within 5° grid cells across Africa; in total there were approximately 1.8 million footprints. Both maps were designed in ArcGIS.

The largest cumulative aboveground biomass gains took place in the Tropical Moist Broadleaf Forests biome of the Congo basin in areas within Equatorial Guinea, Gabon and the Republic of Congo (Fig. 2). The majority of the Tropical Grasslands, Savannas and Shrublands biome shows cumulative aboveground biomass gains across Africa, although between 2010 and 2015, it exhibited a cumulative loss.

These results provide fresh evidence from the new aboveground biomass dataset presented here that Africa’s forests have switched from a net sink of carbon to a net source after 2010.

## Discussion

Previous studies have led to contradictory insights into whether Africa’s ecosystems are a net sink or a net source of carbon. This study provides the first continent-wide, high-resolution assessment of aboveground woody biomass changes in Africa over a decade, revealing a significant transition from a carbon sink to a source between 2010 and 2017. Our analysis, based on satellite-derived aboveground biomass maps validated with field data, highlights the escalating impact of deforestation in tropical moist broadleaf forests as a primary driver of biomass loss. We acknowledge that satellite-derived AGBD has some limitations due to the saturation at high AGBD ranges. However, there are two facts that give us confidence in our conclusions. First, we have adopted a rigorous uncertainty analysis approach and the 95% confidence intervals of the net changes in AGBD shown in Fig. 2 indicate that we can claim with high confidence that the transition from a carbon sink to a source is real. The confidence intervals do not overlap. Second, Table 1 and Figure S7 show that the AGBD values in our study are reasonably similar to independent country-level statistics reported by FAO. For example, for the Democratic Republic of Congo (DRC), which contains by far the most of the African AGB, the deviation of our estimate from the FAO estimate is only 11%. Furthermore, as most deforestation occurs in the dense moist broadleaf forests where biomass is high, the underestimations we detect beyond 350 Mg/ha suggest that our analysis may not reflect the full impacts of forest loss on carbon emissions and that the reversal from sink to source we suggest may be even more pronounced than what we report here. While gains in savanna regions, potentially due to shrub encroachment, partially mitigated these losses, they are insufficient to reverse the overall trend.

**Table 1:** Percentage difference of AGB relative to FAO, i.e. 100 x (study value—FAO national value)/FAO national value, of this study and the **5** published studies of Saatchi et al.^5^, Baccini et al.^6^, Avitabile et al.^7^, Bouvet et al*.*^30^ and Santoro et al.^31^ for the 6 countries with largest AGB stocks according to FAO. The AGB stocks (Tg) reported by FAO are given in brackets following the country name.

Country	This study	Bouvet et al. 2018	Saatchi et al. 2011	Baccini et al. 2012	Avitabile et al. 2016	Santoro et al. 2020
DRC (40,915)	11	− 10	− 7	11	2	− 18
Angola (9,135)	− 31	− 36	− 24	8	− 53	− 35
Rep. of Congo (7,163)	8	− 2	− 7	− 3	19	− 13
Central African Republic (5,960)	19	− 10	− 15	19	− 38	− 3
Gabon (5,646)	53	25	27	− 3	64	10
Cameroon (5,617)	43	26	37	35	60	16

Our study focuses on aboveground woody biomass stock and its changes, as this is the only pool that is persistent over long time periods and that we can realistically quantify using remote sensing techniques. Although also persistent, soil carbon stocks cannot be estimated with any confidence from remote sensing. At continental and biome scale, we consider carbon fluxes from woody debris and litter as short-term (annual turnover time), with no substantial dampening or enhancing effects on ecosystem-level carbon fluxes.

The implications of this shift are profound. Africa’s forests and woodlands have historically served as a carbon sink. Now, they are contributing to widening the global greenhouse gas emissions gap that needs to be filled to stay within the goals of the Paris Agreement. The significance of this finding to global climate policies and the current revision of the Nationally Determined Contributions to the Paris Agreement is that even more greenhouse gas emission reductions are needed than was the case before this natural carbon sink shut down. Our findings underline the urgent need for strengthened conservation policies, improved forest governance, and targeted restoration initiatives, such as REDD+. The fine-scale spatial and temporal insights provided in this study can support policymakers and scientists in prioritizing interventions to halt biomass loss and enhance carbon sequestration.

New insights into the geographical regions where significant biomass changes have occurred are also presented. The biomass maps at continental and biome scales were validated with a large independent dataset of field plot data and LiDAR-based estimates of aboveground biomass density (Supplementary Material). The fine spatial resolution allowed us to account for the trends in biomass loss associated with forest disturbances as small as one hectare, which was not possible until recently^9,17,31,32^.

Total aboveground biomass stocks of African forests and woodlands are estimated at of 118 Pg biomass, which equates to approximately 59 Pg carbon. These overall values are consistent with previous estimates using independent datasets, e.g. 113 Pg^5^, 111 Pg^9^, 116 Pg^8^, and 129 Pg^6^ biomass stocks, but it is higher than the estimates provided by Santoro et al.^31^ and Avitabile et al.^7^ (85 Pg and 96 Pg, respectively). The latter two studies excluded some areas with woody aboveground biomass by applying a spatial forest mask to exclude several woody vegetated areas, such as savannas, that did not fit their specific forest definitions, while our estimates include all woody vegetation in the tropical forest and savanna biomes with a percentage tree cover above 1%.

The aboveground biomass estimates presented here, and other Earth observation-based studies disagree with official biomass statistics for some countries at national level as reported by the United Nations Food and Agriculture Organisation (FAO) (Table 1). These differences are most likely also the consequence of using different national forest definitions^33,34^ leading to substantial differences in the reported total national forest area.

The pantropical and continental AGBD maps products derived by Saatchi et al.^5^, Baccini et al.^6^, Avitabile et al.^7^, Bouvet et al.^35^ present spatial resolutions from 25 m to 1 km, and reported Rel. RMSD = 33%, RMSD = 39 Mg/ha, RMSD = 87–90 Mg/ha, and RMSD = 17–19 Mg/ha for Africa, respectively. However, the later African AGBD 25m pixel map is constrained to AGBD levels below 100 Mg ha^-1^. Only the global map from Santoro et al.^31^ presents the same spatial resolution as the current study (i.e. 100m pixel size) but reports larger errors with Rel. RMSD = 57–73% for tropical and subtropical regions compared to this study’s Rel. RMSD = 33–40%.

The mean annual net aboveground biomass change shows gains of +57 ± 9 Tg yr^-1^ for the period 2007 to 2017. However, analysing the gains and losses for different time periods, an annual net gain of +439 ± 66 Tg yr^-1^ is found in the earlier time period from 2007 to 2010, followed by a net loss of -106 ± 16 Tg yr^-1^ from 2011 to 2017. This indicates a rapid acceleration of aboveground biomass losses in Africa, which is driven by increasing biomass loss rates in the Tropical Moist Broadleaf Forests.

The evidence presented here suggests that Africa’s forests and woodlands have switched from a net carbon sink into a net source because of increased biomass losses due to human activities and natural disturbances. This finding is consistent with growing forest loss rates in Africa from 2010 onwards as reported by FAO^36^, and the increasing forest cover loss rates based on satellite observations from 2012 to 2013 onwards by Hansen et al.^29^. FAO^36^ statistics on increasing annual harvested roundwood from 277 million m^3^ in 1961 up to 768 million m^3^ by 2017 also confirm these forest losses. The observed trends may be further exacerbated in the future by population growth in Africa^37^, the increasing export demand particularly from Asia^38^, and the resulting pressure on natural resources (agricultural expansion for commodity crop, timber and fuelwood). The long-term persistence of these trends will depend on local governance and whether resources are used sustainably.

The results provide further independent evidence of a shift in forest functioning from a carbon sink to a source around this time period, which is consistent with recent studies of all pantropical regions^19,35,39,40^.

A comprehensive assessment of the terrestrial greenhouse gas budget of Africa found that most studies agree that Africa is a small sink of carbon on an annual scale, with an average value of − 0.61 ± 0.58 Pg C yr^−1^^4^. The net loss of aboveground woody biomass from 2011 to 2017 of − 106 ± 16 Tg yr^-1^ observed here (weighted mean) may well tip the overall carbon budget into a net source overall when considering all carbon pools and fluxes.

The world needs to step up efforts to protect the substantial carbon stocks in Africa’s aboveground woody biomass and restore lost forest areas to counter the climate crisis, as agreed in the Glasgow Leaders Declaration on Forests and Land Use^41^ at the 26^th^ Conference of the Parties (COP26) to halt net deforestation by 2030. The world otherwise risks losing an important carbon sink needed to achieve the goals of the Paris Agreement. Reversing biomass losses in Africa requires actions in the political, economic and societal spheres, to promote capacity building^42^, improve forest governance^43^, implement financial incentives through the REDD+ initiative^44^, and facilitate technological infrastructure, such as satellite-enabled forest alert systems to halt illegal logging such as those deployed in Kenya^45^. Restoration initiatives such as AFR100^46^ and Restor^47^ will also be needed.

Future research should explore the underlying drivers of regional variability in biomass dynamics and assess the potential of emerging technologies for near-real-time monitoring of forest disturbances and strengthening forest governance.

## Methods

Assessing forest aboveground biomass dynamics over long time periods and at continental to global scale requires reference observations such as forest inventory field plots in combination with satellite Earth observation. To overcome the low availability and quality of reference data, we used the spaceborne Geoscience Laser Altimeter System (GLAS) onboard the Ice, Cloud, and land Elevation Satellite (ICESat)^48^, which operated from 2003 to 2010 and acquired millions of Light Detection and Ranging (LiDAR) footprints, providing measurements of canopy height and other biophysical metrics relating to canopy structure that are highly correlated with aboveground biomass^49^. The Global Ecosystem Dynamics Investigation (GEDI) LiDAR instrument^22^ onboard the International Space Station that commenced operation in 2019 uses similar technology to the GLAS/ICESat instrument but with smaller footprints and much denser coverage (narrower spacing between footprint locations along the orbit), providing a dense network of training and validation data on forest canopy height.

Based on a machine learning algorithm, L-band Synthetic Aperture Radar (SAR) backscatter image mosaics acquired by ALOS PALSAR-1/PALSAR-2^48^ and optical multispectral Landsat-derived percent tree cover maps^29^ were used as joint predictors to extend the canopy height obtained from GEDI to canopy height maps across the whole of Africa. Regional maps of aboveground biomass density derived from airborne LiDAR over a range of biomes in Africa were then used to derive an empirical model that estimates aboveground biomass density as a function of canopy height. The LiDAR-based aboveground biomass footprint estimates from LiDAR were used to train the machine learning model. The model was then used to produce annual Africa-wide maps of aboveground biomass density and its standard deviation (SD) at 100 m pixel spacing for the period 2007 to 2017. The maps were validated using a large independent dataset of field plot measurements across the continent. The aboveground biomass density maps and associated standard deviations were used to estimate the African aboveground woody biomass stock and its annual changes (with confidence intervals based on the uncertainty characterization described in Supplementary Materials) with the aim of contributing to improvement of carbon inventories, understanding trends, and testing whether the aboveground biomass change rate has increased, reduced or changed sign over the period 2007-2017.

### Datasets

*Spaceborne LiDAR from GEDI*. The GEDI L2B Canopy Cover and Vertical Profile Metrics product (version 1), available from the NASA/USGS Land Processes Distributed Active Archive Center^50^, was collected from April 2019 to June 2019 (Fig. 3a). The LiDAR metrics estimated by this product are representative of a 25 m diameter footprint on the ground. Version 1 of the product has a geolocation error of approximately 15-20 m, so can be difficult to use with moderate spatial resolution imagery such as Sentinel-1/-2 or Landsat (i.e. 10-30 m spatial resolution pixels).

*Airborne laser scanning (ALS)*. This study uses gridded LiDAR-derived aboveground biomass density maps based on airborne LiDAR acquired in 2016 at 4 different sites in Gabon (Lope, Mabounie, Mondah, and Rabi)^51,52^, and in 2015-16 at 2 sites in Kenya (Taita Hills and Maktau)^53,54^. The canopy height to AGBD model was developed using 50% of the pixels from these datasets, while the remaining 50% were used to validate the aboveground biomass product.

*Field plot data of aboveground biomass density*. We collated a dataset consisting of 10,837 aboveground biomass density reference field plots across Africa (see Table S2 in supplementary material) to be used as an independent validation dataset for the 2017 map (Fig. 3). The assessment was limited to 2017 due to the lack of re-measured plot data. Field plots were measured in different years, mainly between 2000 and 2017, with the vast majority before 2010. The plot data are from different national forest inventories and research projects, so have various plot designs, sizes and shapes. Since most of the plots do not have accurate spatial coordinates due to licensing restrictions, we cannot use them directly to validate the 100 m resolution pixel maps. Instead, we followed the approach described by Santoro et al.^31^ and Araza et al.^15^ in which the plot values were first adjusted to minimize the temporal and areal mismatches between the plot and map estimates of aboveground biomass density. The adjustment was necessary because of the uneven spatial distribution of the reference samples, the variety of plot sizes used, variations in field survey methods, and used allometric equations to estimate biomass of the plots^55^. The plots were mostly smaller than 1 ha and often represented only a small fraction of the area covered by a 1 ha biomass map pixel. To reduce the effect of random errors caused by different resolutions of the reference dataset and the biomass map, we aggregated the map and the plot data to 0.1° grid cells^15^. This procedure yielded 463 grid cells with reference aboveground biomass density values for validation (Fig. 3b). The standard deviation associated with each of these values was estimated by accounting for the principal plot measurement error sources (as described in Araza et al.^15^).

*ALOS PALSAR/ALOS-2 PALSAR-2 radar image mosaics*. JAXA’s annual mosaics of L-band SAR HH and HV polarised backscatter (γ^0^) were based on ALOS PALSAR from 2007 to 2010 and ALOS-2 PALSAR-2 from 2015 to 2017^56,57^. No mosaics are available for 2011 to 2014. The PALSAR-2 mosaics for 2018 to 2020 are available, but the pre-processing and geolocation approaches are different from the previous mosaics, so they were excluded from this analysis. The mosaics are a calibrated, 16-look, re-projected, orthorectified and slope-corrected product with 25 m pixel spacing, to which a de-striping process has been applied^22,31,33^. The PALSAR and PALSAR-2 mosaics were normalised to reduce artefacts and to ensure temporal consistency of the radar backscatter signal, allowing the same trained model to be used for the whole time series. Artefacts in the mosaics usually result from changes in moisture conditions between image acquisitions, which affects the backscatter, or appear in the pre-processing due to inadequate calibration and/or topographic corrections. We followed a similar approach to that described in^58^, but instead of superpixels used a circular moving window 100 pixels in diameter (~2,000 ha). This normalises the PALSAR/PALSAR-2 imagery to a common baseline based on the mean and standard deviation of backscatter of the PALSAR mosaics (2007-2010). Implicit in this procedure is the assumption that continuous changes with scale larger than 2,000 hectares did not occur from year to year. Normalizing the images at this large scale also tends to ensure that local changes due to disturbances and vegetation growth are preserved. The analysis used both HH and HV polarisations and two additional metrics, the Cross-polarisation Ratio (*CpR* = *HH⁄HV*) and the Radar Forest Degradation Index (*RFDI* = *(HH–HV)⁄(HH*+*HV)*)^59^.

*Percent tree cover data*. A 30 m Landsat-based map product of percent tree canopy cover for the year 2000 and annual tree cover loss estimates for the period from 2000 to 2017^29^ was used to generate annual percent tree cover maps for each year. For the year 2007, all pixels detected as forest cover loss were set as 0% percent tree cover, while pixels detected as having forest cover loss in previous years (i.e. from 2000 to 2006) were set to “no data”, as we have no information on regrowth after the disturbance. The canopy height and aboveground biomass predictions for these “no data” pixels were performed using only PALSAR as a predictor variable (see Modelling Framework). We repeated this process for all the years within our study period (2007-2010 and 2015-2017). PALSAR data and Landsat percent tree cover datasets were mosaicked and co-registered to generate two stacks of predictor datasets, with 50 m and 100 m pixel spacing, respectively. Woody vegetated areas were defined as pixels with equal or above 1% tree cover.

### Modelling framework

*GEDI footprint selection and clustering*. We used the maximum footprint height in a footprint, provided by the GEDI L2B footprint product, as reference canopy height metric, but performed a filtering process to select only the highest quality footprints for training and validation purposes. Coverage footprints were excluded due to their lack of laser light penetration in dense forests and only footprints acquired by the full power beams were used. Only night acquisitions (solar elevation < 0°) with a beam sensitivity greater than 95% were retained. Low quality footprints, as indicated by the L2B quality assurance layers, were discarded, as were footprints with canopy height above the 99.9^th^ percentile of the initial set. The Copernicus Global Land Cover dataset^60^ was used to exclude footprints in non-vegetated classes. We used the 11 forest classes and the shrubland class for this purpose^60^. After the filtering, approximately 1.8 million footprints were available, distributed across the African continent (Fig. 3). The GEDI footprints were grouped into 4-footprint clusters along the track direction, in each of which the top canopy height values (RH_100_) were averaged to correct for sampling and geolocation errors. This provided the main reference data for training and validating our canopy height model. The average by cluster increases the sampled area of our reference unit from 0.05 ha (1 footprint) to 0.2 ha (4 footprints) and has been demonstrated to increase accuracy when training models with spaceborne LiDAR footprints^6^. The larger sampled area helps to average out various errors typical of small sampling units (e.g. small inventory plots), such as sampling error and geolocation error. Only clusters with 4 consecutive footprints were used.

**Fig. 4:**
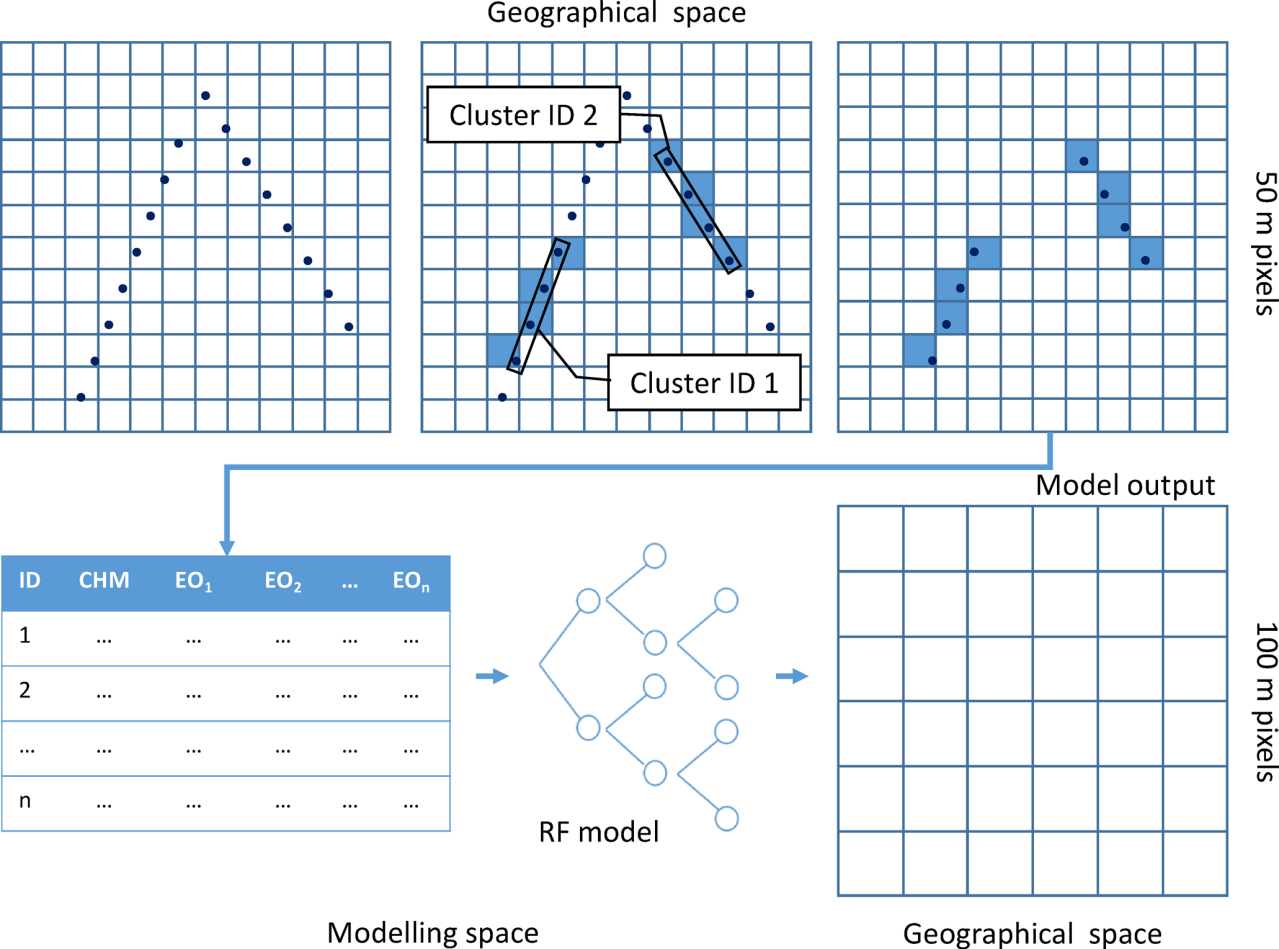
Modelling scheme following the 2-scale approach showing the connection between GEDI footprint clusters and EO datasets in geographical space, their relationship in modelling space, and the model output in geographical space. Pixel signatures values from the Earth observation predictors (% Tree Cover, PalSAR HH and HV, CpR and RFDI) and GEDI top canopy heights are averaged for each cluster before training the random forest (RF) model.

*Canopy height modelling*. We used a non-parametric machine learning Random Forests (RF) regression algorithm^61^, following the same framework as in^62^ and ^63^, to generate a canopy height model (CHM) and its associated error at pixel level using PALSAR/PALSAR-2 radar backscatter and Landsat Percent Tree Cover for the same years as predictors. We used 100 trees for each RF model run. Generation of the canopy height model uses two different resolutions (Fig. 4): (i) Remote sensing signatures were extracted from the four 50 m pixels that overlap the 0.2 ha area corresponding to each cluster of four GEDI footprints and averaged; these four pixels are equivalent to the area of 1 ha. (ii) The model training and the prediction output are at 1 ha pixel size, i.e. 100 m by 100 m.

The conversion from 50 m to 100 m was needed because geolocation errors in the GEDI footprint products (15–20 m for version 1 and around 10 m for version 2) prevent small (< 50 m) pixels being adequately matched to single GEDI footprints. Additionally, as with small forest inventory plots, a large tree within the footprint biases the canopy height and makes the value unrepresentative of the canopy height within 1 ha. This two-scale scheme follows Saatchi et al.^5^ and Baccini et al.^6^, and aims to average out these errors by combining several footprints. We assume that 4 footprints located within 1 ha (four 50m x 50m pixels) are more representative of the actual canopy height than 1 or 2 footprints located within a 100 m pixel.

The GEDI canopy height dataset was randomly partitioned into 2 datasets: 10% for training the CHM model (36,944 GEDI clusters) and the remaining 90% (328,243 clusters) for independent validation. Training was performed within a jack-knife/k-fold framework in which the training reference data (10% of the original dataset) were spatially partitioned into k subsamples with k = 10. The jack-knife / k-fold allocation was applied spatially to avoid over-optimistic accuracy metrics due to the possible spatial autocorrelation of the training data^64^. We used a very large dataset for independent validation (i.e., 90% of the original dataset) to avoid a large proportion of the validation dataset being autocorrelated with the training data. Hence, we divided our training dataset (36,944 GEDI clusters) over the whole of Africa into 10 regions with approximately the same number of footprint clusters. A single spatial subsample was kept aside as the validation data for testing the RF model, while the remaining k-1 spatial subsamples were used as training data. The cross-validation process was then repeated k times. Thus, 10 canopy height maps were generated. The mean value of all 10 predictions for each pixel was then used as the final canopy height estimate, and their standard deviation as the prediction error. We also propagated the GEDI footprint height measurement error, the sampling error and the error arising from the temporal difference between GEDI footprints and the satellite imagery to estimate the total error of our canopy height maps (see Supplementary Material).

We trained 2 different random forest (RF) models to predict canopy height. The first one (main model) used annual percent tree cover and SAR backscatter data, while the second one only used SAR backscatter data. Our main canopy height model uses L-band PALSAR/PALSAR-2 radar imagery together with the Landsat percent tree cover product. This model was applied for all pixels from the year 2000 to the given year that were undisturbed according to Hansen et al.^29^. However, for the pixels detected as disturbed (i.e., forest cover loss), a second canopy height model based only on PALSAR/PALSAR-2 was fitted and used for the years after the disturbance, as this is better suited to detecting any recovery (the Landsat Percent Tree Cover product assumes no regrowth after a forest loss event).

*Conversion of canopy height to aboveground woody biomass density*. We developed a single empirical linear model to estimate the square root of aboveground biomass density as a function of canopy height. The model was based on six airborne LiDAR aboveground biomass density maps covering closed-canopy moist tropical forest, mangrove, montane forest, drylands and open savannas (see supplementary material).

1 $$\sqrt {{\text{AGBD}}} = \beta_{0} + \beta_{1} \cdot {\text{CHM}} + \varepsilon_{i}$$

The square root of AGBD is derived to reduce heteroscedasticity^65^. Conversion to AGBD requires correction of the inherent negative bias in the square root transformation^66^ using the ratio estimator proposed by Snowdon et al.^67^:

2 $${\text{AGBD}} = (\beta_{0} + \beta_{{1}} \cdot {\text{CHM}})^{{2}} \cdot {\text{ratio}}\,{\text{estimator}}$$

*Boveground biomass density change analysis*. For a given pixel, we identified a significant biomass loss between times t1 and t2 if *AGBD*_*t1*_*–SD*_*t1*_ > *AGBD*_*t2*_ + *SD*_*t2*_*,* while there is a significant biomass gain if *AGBD*_*t1*_ + *SD*_*t1*_ < *AGBD*_*t2*_*–SD*_*t2*_, where the AGBD values at t1 and t2 are *AGBD*_*t1*_ and *AGBD*_*t2*_ respectively, and their corresponding SD values are *SD*_*t1*_ and *SD*_*t2*_. Changes between consecutive years were considered significant when the error bounds on the estimates of AGBD at the two times did not overlap, otherwise the measured change was considered insignificant. For pixels showing a significant annual gain or loss, we calculated the cumulative significant change over the time series. If this was negative at the end of the period (i.e. 2017), we accepted it as significant biomass loss, and assign this loss value to the year with the highest loss (when significant loss is detected in two or more consecutive pairs of images). Biomass losses usually result from forest cover loss events, but could also arise from other causes, such as natural or anthropogenic fires. A similar approach was taken to biomass gains. However, small gains and losses are hard to detect using this method, and biomass gains were very scarce in forest areas with high aboveground biomass density (i.e. mature forest). Either change is negligible in such areas due to a balance between mortality and growth, or it simply cannot be detected due to insufficient sensitivity of the Earth observation signal when biomass is high.

For pixels that were undisturbed (i.e. with positive cumulative significant biomass change or non-significant biomass change), the slope of the temporal linear regression for the given time period was used to represent the average rate of vegetation growth or progressive loss. We calculated the long-term Sen’s slope^68^ and only accepted significant slopes (*p *< 0.05). Additionally, we followed Xu et al.^24^ in assuming that biomass gain cannot be properly measured once aboveground biomass density exceeds a certain level, as evidenced by the steep increase in the standard deviation of our estimates for high aboveground biomass density (Fig. S5). Thus, for undisturbed pixels with aboveground biomass density above 50 Mg ha^-1^ we used the IPCC aboveground biomass density growth factors for mature forest^69^.

Once all the significant changes across the time-series were identified, we generated a new aboveground biomass density dataset using the aboveground biomass density map from 2017 as reference (the year used to train the model, equation^1^), and then rolled only the significant changes back to 2007 to generate a consistent temporal dataset.

*Biome-level AGB quantification*: We used terrestrial biomes as spatial units to calculate regional AGB statistics. Biomes are not strictly defined by the physical land cover types (e.g. forests, shrublands etc.), but rather a complex combination of climate and vegetation conditions^70^. While biome extents may be dynamic, due to gains and losses in woody vegetation across landscapes, alongside changes in regional climatic conditions, to our knowledge, there is no data product that accounts for land cover dynamics to delineate biomes on an annual basis. We therefore used the most up-to-date static product available, the Ecoregions2017 Resolve biome map^14^ and assumed that biome extents have not significantly changed over the study period. Quantifying woody aboveground biomass losses / gains is itself an indicator of biomass changes in forests and shrublands.

## Supplementary Information

Below is the link to the electronic supplementary material.

Supplementary Material 1

## Data Availability

The datasets used and/or analysed during the current study will be available from the corresponding author on reasonable request.
