# Supplementary material for: Loss of tropical moist broadleaf forest has turned Africa’s forests from a carbon sink into a source
Source: Sci Rep. 2025 Nov 28;15:41744. doi: 10.1038/s41598-025-27462-3 (PMC12663118; doi:10.1038/s41598-025-27462-3)
Supplement: Supplementary file 1 — Supplementary Material 1 [file 41598_2025_27462_MOESM1_ESM.docx]

# Title: Loss of tropical moist broadleaf forest has turned Africa’s forests from a carbon sink into a source

# Supplementary Material

## Canopy height modelling

A random forest regression algorithm was trained to estimate canopy height and cross-validated within a jack-knife / k-fold framework, with k = 10. We optimised the number of decision trees in the random forest model within this framework by starting with 300 trees x 10 folds = 3000 trees in total and progressively reduced the number of trees. The optimal number of trees in terms of performance and computational demand was around 50 trees x 10 folds = 500 trees in total, since for more than 50 trees the performance was almost unchanged. The relative variable importance in the model was analysed as the relative contribution of each variable to the random forest model predictions. ALOS-2 PALSAR-2 HV polarization radar backscatter and Landsat Percent Tree Cover (PTC) each contributed about 24%, while the contribution of the other variables ranged from 16% to 20% (Fig.S1). The overall contribution of the SAR predictors to the estimation of canopy height was 76%.


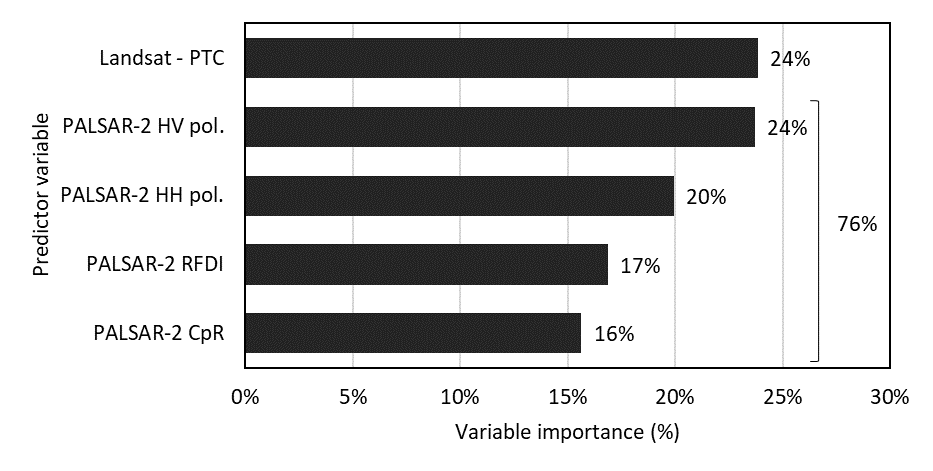


**Fig.S1** | Variable importance analysis for the spatial 10-fold RF model. Predictors included were the Landsat Percent Tree Cover (PTC), PALSAR-2 cross-polarization (HV pol.), vertical polarization (HH pol.), Radar Forest Degradation Index (RFDI) and the Cross-polarization Ratio (CpR)

We tested a random sampling and a spatial sampling approach to generate our 10 folds. We adopted the spatial sampling framework as the results were slightly more conservative and this mitigated potential spatial autocorrelation issues when reporting the metrics (Table S1 and Fig.S2). Canopy height was also validated against the large independent dataset of 4-footprint GEDI clusters (90% of the initially available data) (Table S1).

The accuracy of the canopy height maps is expressed in terms of differences, not errors, since the reference aboveground biomass density data from LiDAR and field plots inevitably contain some errors. We calculated the mean bias difference (MBD), the root-mean-square difference (RMSD), and the Relative RMSD (%) as defined in Rodríguez-Veiga, Carreiras [1].


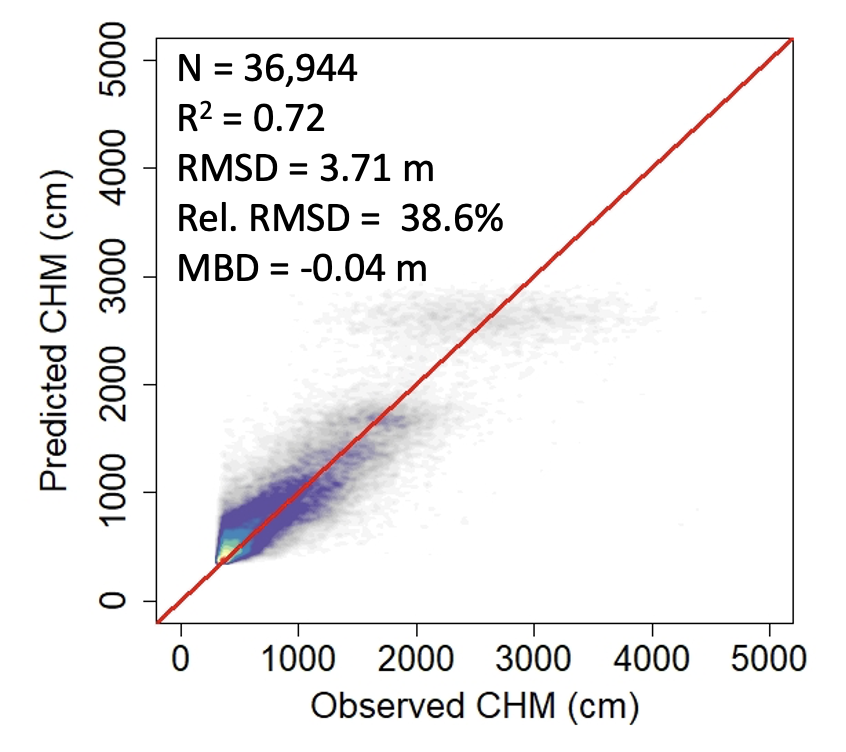


**Fig.S2** | Scatterplot of canopy height (CHM) predictions against observed CHM values using spatial cross-validation. Warmer colours indicate higher point density, and the red solid line corresponds to the identity line.

## Aboveground biomass density modelling

An empirical linear model relating $\sqrt{AGBD}$ to canopy height was developed using the six airborne LiDAR-based aboveground biomass density reference maps. These were gridded to match the canopy height map pixels and aggregated to 300 m resolution to average out random errors. Fig.S3 shows the relationship between the square root of aboveground biomass density and the canopy height model from LiDAR.

**Fig.S3** | Scatterplot of the relationship between the airborne LiDAR-derived $\sqrt{AGBD}$ and the canopy height map (CHM) derived from the random forest model. The fitted model has form $\sqrt{AGBD}$= β_0_ + β_1_·CHM

Independent datasets consisting of airborne LiDAR-based aboveground biomass density estimates and a large dataset of field plot measurements across Africa were used to validate the aboveground biomass density maps. Accuracy assessment was performed at biome level (Table S3) and at continental level by biomass range (Table S4). Comparisons with country-level estimates are shown in Fig.S7.

## Aboveground biomass time series maps

Annual maps of aboveground biomass density were generated for the years 2007 to 2010 and 2015 to 2017 at 100 m spatial resolution, which allowed significant biomass gains and loss to be estimated across the continent. Fig.S4 shows an example of a time series of biomass data for a small area in Gabon. Maps were not generated for 2011 to 2014 due to the lack of PALSAR/PALSAR-2 data for those years.

**Fig.S4** | Example of the time series (2007-2017) of AGBD maps in Ngounié Province (Gabon), overview location map, and the corresponding histograms of AGBD for the given area within the red rectangle by year. The biomass loss event slightly starting in 2016, and fully happening in 2017 skews the corresponding histograms to lower AGB values.

**Fig.S5** | Aboveground Biomass (AGB) time series (Pg) per biome (black lines): a) Tropical Moist Broadleaf Forests, b) Tropical Grasslands, Savannas and Shrublands, c) Montane Grasslands and Shrublands, d) Mediterranean Forests, Woodlands and Scrub e) Deserts and Xeric Shrublands, f) Mangroves, g) Tropical Dry Broadleaf Forests, h) Temperate Conifer Forests, i) Flooded Grasslands and Savannas. Note that a) and b) have a much larger scale on the -axis and that estimates for the individual years between 2011 and 2014 are not calculated since no L-band radar was in orbit over that period; we therefore assume a constant rate of AGB change over that period. Dashed lines correspond to 95% confidence intervals.

## Uncertainty characterization

Confidence intervals for aboveground biomass density map-based estimates over large areas require model-based inferential methods [55]. We assumed a normal distribution to calculate the intervals. Mean square errors (MSE) include residual variability $\hat{Var}_{res}(\hat{AGBD})$and sampling variability $\hat{Var}_{sam}(\hat{AGBD})$ as follows:

$${MSE}_{total}(\hat{AGBD})= \hat{Var}_{res}(\hat{AGBD})+\hat{Var}_{sam}(\hat{AGBD})$$

$${SE}_{total}(\hat{AGBD})=\sqrt{{MSE}_{total}(\hat{AGBD})}$$

The residual variability ($\hat{Var}_{res}(\hat{AGBD})$) at pixel level is calculated following [1, 3, 4]. The total SD (ε_CHM_) for the CHM retrieval at pixel level is propagated as follows:

ε_CHM_ = (ε^2^_measurement_ + ε^2^_temporal_difference_ + ε^2^_local_spatial_variability_ + ε^2^_prediction_)^1/2^

where ε_measurement_ is the SD arising from the measurement of CHM in the GEDI footprint, ε_temporal_difference_ is the SD from the use of GEDI footprints and Earth observation imagery acquired at different time periods, and ε_local_spatial_variability_ is the SD originating from the spatial variability of canopy height within the pixel. The ε_prediction_ corresponds to the model SD originating from the jack-knife / k-fold framework, but ε_prediction_ also accounts for errors that arise if the sampling sites are not truly representative of the distribution of canopy height in the region [4]. The value of ε_measurement_ is assumed to be 50 cm [5] (28). The SD from the local spatial variability is extrapolated from Réjou-Méchain, Muller-Landau [6] as 19.78% when using 0.2 ha plot area (4 footprints) with a 1 ha output pixel area. To account for the temporal difference between the GEDI footprints (i.e. 2019) and the Earth observation imagery (i.e. 2017) we used a 5% error [3]. We then generated a total SD (ε_AGB_) at pixel level for the aboveground biomass density maps (Fig.2 and Fig.S5) as:

ε_AGB_ = (ε^2^_CHM_ + ε^2^_LiDAR_ + ε^2^_model_)^1/2^

where ε_LiDAR_ is the SD from the LiDAR-derived aboveground biomass density maps used as reference and was estimated as 15.9% of the aboveground biomass density per pixel. This was derived from uncertainty characterization and the data provided by [7], and propagates field measurements, tree allometries and model errors. We also propagated the model error (ε_model_) of our empirical model (i.e. AGBD=f(CHM)) by predicting ε_model_ as a function of the aboveground biomass density prediction (i.e. ε_model_ = f(AGBD)) based on the training dataset. The pixel-scale standard deviation (SD) as a function of AGBD is shown in Fig.S6.


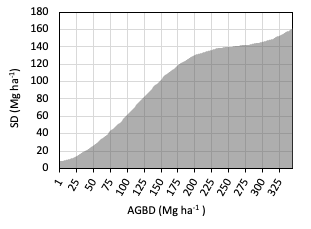


**Fig.S6** | Pixel-scale standard deviation (SD) as a function of the aboveground biomass density (AGBD).

The sampling variability ($\hat{Var}_{sam}(\hat{AGB})$) is calculated using our 10-fold jack-knife framework. We generated 10 different models and biomass predictions from which we estimated the average AGBD, total AGB stocks, and mean square errors (MSE) at biome level and for Africa.

Residual variability can be considered negligible for large areas such as continents or biomes [2, 8], so our estimates at that scale only include the sampling variability. Our precision is reported as ± half the 95% confidence interval. The precision of estimated AGB net stock change (*U*_DAGB_) between two dates was estimated as follows:

$$U_{\Delta AGB}=\sqrt{\frac{{{(U}_{t1}\cdot{AGB}_{t1})}^{2}+{(U_{t2}\cdot{AGB}_{t2})}^{2}}{\left| {AGB}_{t1}+{AGB}_{t2} \right|}}$$

where *U*_DAGB_ is the percentage uncertainty in the subtraction of the quantities (i.e. half of the 95% confidence interval divided by the mean), while *AGB_ti_* and *Ui* are the estimated AGB stocks for the given times and the percentage uncertainties associated with them, respectively [9].

## Country/territory-level estimates

Table S5 shows a statistical analysis of aboveground woody biomass stocks within countries or territories based on boundary data obtained from the United Nations’ Food and Agriculture Organization (FAO). Fig.S7 compares the aboveground biomass data presented in this study with previously published biomass maps from the literature.

**

**Fig.S7** | a) Country-level aboveground biomass stocks (Tg) estimated by this study plotted against the values reported by FAO and the remote sensing based studies of Saatchi, Harris 4, Baccini, Goetz [10], Avitabile, Herold [11], Bouvet, Mermoz [12]. Each circle represents one national-level biomass stock estimate for a given country from Table S5 (N=49). b) The same plot but excluding the Democratic Rep. of the Congo (largest AGB stock). The dashed black line represents the 1:1 line.

# Tables

**Table S1** | Accuracy assessment of the canopy height map for 2017 using the random 10-fold, the spatial 10-fold, and the large independent validation dataset.

| **Dataset** | **Random 10-fold** | **Spatial 10-fold** | **90% set aside** |
| --- | --- | --- | --- |
| **N** | 36,944 (147,776) | 36,944 (147,776) | 328,243 (1,312,972) |
| **R^2^** | 0.72 | 0.72 | 0.69 |
| **RMSD (m)** | 3.67 | 3.71 | 3.82 |
| **Rel. RMSD** | 38.3% | 38.6% | 31.6% |
| **MBD (m)** | -0.05 | -0.04 | -0.43 |

**Table S2** | Summary of aboveground biomass density field measurements used in the accuracy assessment presented in Fig.1d (main text).

| Type | Brief description | Avg. size ± SD (ha) | Nr 0.1° cells (Nr plots) | Biomes | Period | Ref |
| --- | --- | --- | --- | --- | --- | --- |
| Forestry plots | Selected grid cells of 1-km from harmonized forest management plots. Only cells with >4 plots are selected | 99.83 ± 2.88 | 297 (9,137) | Mangroves; Tropical grasslands, savannas and shrublands; Tropical moist broadleaf forest | 2000-2010 | ^13^ |
| Research plots | Forest Observation System research plots | 0.32 ± 0.04 | 17 (384) | Tropical grasslands, savannas and shrublands; Tropical moist broadleaf forest | 2006-2016 | ^14^ |
| NFI | Guinea Bissau NFI | 0.13 ± 0.0 | 12 (32) | Mangroves | 2007-2008 | ^15^ |
| NFI | Kenya NFI | 0.09 ± 0.0 | 11 (143) | Tropical grasslands, savannas and shrublands; Tropical moist broadleaf forest | 2014-2016 | ^1, 16^ |
| NFI | Mozambique NFI | 0.1 ± 0.02 | 124 (1,119) | Mangroves; Tropical grasslands, savannas and shrublands; Tropical moist broadleaf forest | 2011 | ^17^ |
| Research plots | Nigeria mangrove plots | 0.25 ± 0.0 | 2 (22) | Mangroves | 2016-2017 | ^18^ |

**Table S3** | Accuracy assessment of the aboveground biomass density map for 2017 using a large independent field plot dataset for major biomes.

| Biomes | M | TGSS | TMBF |
| --- | --- | --- | --- |
| N | 16 (84) | 284 (6,822) | 163 (3,931) |
| MAD (Mg ha^-1^) | 20.9 | 55.4 | 58.7 |
| Rel. MAD | 20.2% | 28.4% | 20.4% |
| RMSD (Mg ha^-1^) | 31.0 | 72.8 | 79.5 |
| Rel. RMSD | 29.9% | 37.3% | 27.6% |
| MBD (Mg ha^-1^) | -14.4 | 10.6 | 7.1 |
| R^2^ | 0.91 | 0.73 | 0.47 |

*M - Mangroves, TGSS - Tropical grasslands, savannas and shrublands, TMBF - Tropical moist broadleaf forest*

**Table S4a** | Accuracy assessment of the 2017 aboveground biomass density map by biomass range using a large independent field plot dataset.

| AGBD range  (Mg ha^-1^) | N | MAD  (Mg ha-1) | Rel. MAD | RMSD  (Mg ha-1) | Rel. RMSD | MBD  (Mg ha-1) |
| --- | --- | --- | --- | --- | --- | --- |
| 0-25 | 14 (110) | 22.0 | 157% | 36.5 | 259% | 22.0 |
| 25-50 | 36 (340) | 14.2 | 38% | 19.5 | 52% | 4.5 |
| 50-75 | 38 (327) | 17.7 | 28% | 21.2 | 33% | -11.0 |
| 75-100 | 27 (228) | 34.2 | 40% | 37.4 | 44% | -34.2 |
| 100-125 | 16 (124) | 33.6 | 30% | 37.5 | 34% | -33.6 |
| 125-150 | 8 (116) | 73.1 | 54% | 79.9 | 59% | 26.2 |
| 150-175 | 10 (161) | 71.7 | 44% | 87.4 | 54% | 1.1 |
| 175-200 | 11 (179) | 104.0 | 55% | 120.2 | 63% | 62.7 |
| 200-225 | 20 (492) | 99.6 | 47% | 108.0 | 50% | 47.8 |
| 225-250 | 45 (1406) | 96.2 | 40% | 103.9 | 44% | 73.0 |
| 250-275 | 42 (1477) | 77.6 | 29% | 85.2 | 32% | 48.9 |
| 275-300 | 51 (1512) | 60.0 | 21% | 70.6 | 25% | 15.7 |
| 300-325 | 50 (1616) | 55.0 | 18% | 64.8 | 21% | 7.6 |
| 325-350 | 40 (1313) | 39.8 | 12% | 56.4 | 17% | -4.9 |
| 350-375 | 29 (884) | 35.7 | 10% | 66.3 | 18% | -21.7 |
| 375-400 | 11 (267) | 37.8 | 10% | 54.8 | 14% | -35.9 |
| >400 | 15 (285) | 120.2 | 27% | 158.7 | 35% | -120.2 |

**Table S4b** | Accuracy assessment of the aboveground biomass density map by biomass range using a large airborne lidar-derived AGBD dataset.

| AGBD range  (Mg ha^-1^) | N | MAD  (Mg ha-1) | Rel. MAD | RMSD  (Mg ha-1) | Rel. RMSD | MBD  (Mg ha-1) |
| --- | --- | --- | --- | --- | --- | --- |
| 0-25 | 1044 | 10 | 60% | 14.3 | 89% | 7.0 |
| 25-50 | 1393 | 14 | 38% | 18.6 | 51% | 6.5 |
| 50-75 | 509 | 19 | 31% | 28.9 | 48% | 5.0 |
| 75-100 | 242 | 31 | 35% | 41.1 | 47% | 2.7 |
| 100-125 | 130 | 40 | 36% | 56.4 | 50% | 5.1 |
| 125-150 | 86 | 54 | 40% | 76.1 | 56% | 7.5 |
| 150-175 | 80 | 63 | 38% | 78.3 | 48% | -2.2 |
| 175-200 | 57 | 68 | 36% | 82.8 | 44% | 9.9 |
| 200-225 | 57 | 71 | 33% | 84.8 | 40% | 18.5 |
| 225-250 | 87 | 69 | 29% | 80.5 | 34% | 41.6 |
| 250-275 | 140 | 62 | 24% | 73.5 | 28% | 23.2 |
| 275-300 | 194 | 51 | 18% | 62.0 | 21% | 7.7 |
| 300-325 | 237 | 49 | 16% | 60.4 | 19% | -4.5 |
| 325-350 | 204 | 58 | 17% | 74.5 | 22% | -40.1 |
| 350-375 | 165 | 53 | 15% | 74.3 | 21% | -46.7 |
| >375 | 159 | 97 | 24% | 116.7 | 29% | -96.9 |

**Table S5** | Summary of aboveground biomass (AGB) stocks by country estimated in this study, in other studies using remote sensing, and the values reported by the UN Food and Agriculture Organization (FAO) in the Forest Resources Assessments (FRA) 2010 [19]. Note that we estimated AGB in this study for all pixels with percent tree cover ≥ 1% according to Hansen, Potapov [20], but other studies in this table do not use the same forest/non-forest mask in the calculation.

| **Country/Territory** | **Aboveground Biomass Stocks (Tg)** | | | | | | |
| --- | --- | --- | --- | --- | --- | --- | --- |
|  | **This study** | **Bouvet et al. 2018** | **Saatchi et al. 2011** | **Baccini et al. 2012** | **Avitabile et al. 2016** | **Santoro et al. 2020** | **FRA 2010** |
| **Algeria** | 175.1 | 439.6 |  |  |  | 96.7 | 145.8 |
| **Angola** | 6,286.5 | 5,804.2 | 6,920.8 | 9,879.2 | 4,252.1 | 5,916.0 | 9,135.4 |
| **Benin** | 304.7 | 375.0 | 337.5 | 441.7 | 68.8 | 194.2 | 547.9 |
| **Botswana** | 261.4 | 702.1 | 685.4 |  | 272.9 | 136.3 | 1,345.8 |
| **Burkina Faso** | 246.7 | 522.9 | 314.6 | 304.2 | 91.7 | 74.9 | 608.3 |
| **Burundi** | 74.9 | 106.3 | 152.1 | 154.2 | 45.8 | 25.4 | 35.4 |
| **Cameroon** | 8,081.6 | 7,068.8 | 7,708.3 | 7,604.2 | 9,004.2 | 6,518.4 | 5,616.7 |
| **Central African Republic** | 7,097.4 | 5,381.3 | 5,058.3 | 7,091.7 | 3,670.8 | 5,771.5 | 5,960.4 |
| **Chad** | 627.6 | 1,060.4 |  |  |  | 272.0 | 1,322.9 |
| **Côte d'Ivoire** | 2,037.6 | 1,966.7 | 2,302.1 | 2,670.8 | 1,216.7 | 1,008.0 | 3,837.5 |
| **Democratic Republic of the Congo** | 45,368.4 | 36,970.8 | 37,906.3 | 45,556.3 | 41,595.8 | 33,624.4 | 40,914.6 |
| **Djibouti** | 0.1 | 62.5 | 14.6 | 10.4 | 16.7 | 0.0 |  |
| **Egypt** | 49.2 | 143.8 |  |  |  | 0.0 | 14.6 |
| **Equatorial Guinea** | 920.3 | 658.3 | 756.3 | 527.1 | 945.8 | 638.5 | 422.9 |
| **Eritrea** | 9.4 | 239.6 | 110.4 | 83.3 | 70.8 | 2.6 |  |
| **Ethiopia** | 2,799.6 | 4,708.3 | 4,172.9 | 3,775.0 | 1,712.5 | 1,819.7 | 456.3 |
| **Gabon** | 8,666.5 | 7,035.4 | 7,183.3 | 5,466.7 | 9,277.1 | 6,228.3 | 5,645.8 |
| **Gambia** | 16.5 | 66.7 | 18.8 | 29.2 | 4.2 | 10.6 | 66.7 |
| **Ghana** | 1,055.0 | 1,087.5 | 1,252.1 | 1,416.7 | 677.1 | 492.9 | 793.8 |
| **Guinea** | 1,431.2 | 2,629.2 | 1,681.3 | 1,781.3 | 487.5 | 135.5 | 1,289.6 |
| **Guinea-Bissau** | 198.0 | 195.8 | 183.3 | 218.8 | 52.1 | 1,064.1 | 200.0 |
| **Kenya** | 859.8 | 2,035.4 | 1,675.0 | 1,081.3 | 568.8 | 422.8 | 991.7 |
| **Lesotho** | 21.8 | 27.1 | 89.6 |  | 31.3 | 12.9 | 4.2 |
| **Liberia** | 1,883.5 | 1,829.2 | 2,089.6 | 1,883.3 | 2,441.7 | 998.8 | 1,218.8 |
| **Libya** | 2.7 | 85.4 |  |  |  | 0.8 | 12.5 |
| **Madagascar** | 2,703.0 | 4,043.8 | 3,970.8 |  | 2,504.2 | 3,578.9 | 3,387.5 |
| **Malawi** | 249.8 | 318.8 | 454.2 | 560.4 | 158.3 | 152.5 | 300.0 |
| **Mali** | 447.2 | 868.8 |  |  |  | 167.9 | 587.5 |
| **Mauritania** | 1.4 | 83.3 |  |  |  | 0.5 | 14.6 |
| **Morocco** | 116.0 | 541.7 |  |  |  | 40.6 | 464.6 |
| **Mozambique** | 3,431.3 | 4,091.7 | 4,795.8 |  | 1,852.1 | 2,166.4 | 3,525.0 |
| **Namibia** | 252.8 | 1,295.8 | 585.4 |  | 350.0 | 144.4 | 437.5 |
| **Niger** | 11.2 | 177.1 |  |  |  | 1.0 | 77.1 |
| **Nigeria** | 2,645.5 | 3,864.6 | 3,275.0 | 3,447.9 | 1,406.3 | 1,768.5 | 2,260.4 |
| **Republic of Congo** | 7,757.4 | 6,995.8 | 6,685.4 | 6,943.8 | 8,537.5 | 6,261.4 | 7,162.5 |
| **Rwanda** | 88.1 | 127.1 | 150.0 | 152.1 | 64.6 | 39.7 | 81.3 |
| **Senegal** | 226.1 | 406.3 | 400.0 | 377.1 | 100.0 | 147.1 | 708.3 |
| **Sierra Leone** | 643.7 | 575.0 | 720.8 | 847.9 | 447.9 | 273.1 | 450.0 |
| **Somalia** | 326.5 | 2,060.4 | 827.1 | 493.8 | 516.7 | 66.5 | 820.8 |
| **South Africa** | 1,027.4 | 1,727.1 | 3,554.2 |  | 1,027.1 | 572.2 | 1,681.3 |
| **South Sudan** | 1,850.4 | 1,914.6 | 2,039.6 | 2,885.4 | 481.3 | 1,195.3 |  |
| **Sudan** | 399.0 | 1,083.3 |  | 658.3 |  | 481.0 | 2,902.1 |
| **Kingdom of eSwatini (formerly Swaziland)** | 56.4 | 64.6 | 97.9 |  | 31.3 | 40.6 | 45.8 |
| **Tanzania** | 3,347.7 | 3,750.0 | 4,187.5 | 5,645.8 | 1,666.7 | 2,171.2 | 4,206.3 |
| **Togo** | 169.5 | 158.3 | 225.0 | 260.4 | 54.2 | 132.2 |  |
| **Tunisia** | 43.4 | 118.8 |  |  |  | 22.4 | 18.8 |
| **Uganda** | 759.4 | 877.1 | 1,031.3 | 1,231.3 | 452.1 | 346.5 | 227.1 |
| **Zambia** | 2,964.1 | 3,420.8 | 4,345.8 | 5,866.7 | 2,108.3 | 1,917.8 | 5,033.3 |
| **Zimbabwe** | 812.9 | 1,264.6 | 1,591.7 | 1,395.8 | 320.8 | 211.9 | 1,025.0 |
| **Total Africa** | 118,806 | 121,032 | 119,550 | 120,742 | 98,584 | 87,365 | 116,004 |

## References

## 1. Rodríguez-Veiga P, Carreiras J, Smallman TL, Exbrayat J-F, Ndambiri J, Mutwiri F, et al. Carbon Stocks and Fluxes in Kenyan Forests and Wooded Grasslands Derived from Earth Observation and Model-Data Fusion. Remote Sensing 2020, 12(15): 2380.

## 2. McRoberts RE, Næsset E, Saatchi S, Quegan S. Statistically rigorous, model-based inferences from maps. Remote Sensing of Environment 2022, 279: 113028.

## 3. Weisbin CR, Lincoln W, Saatchi S. A Systems Engineering Approach to Estimating Uncertainty in Above‐Ground Biomass (AGB) Derived from Remote‐Sensing Data. Systems Engineering 2014, 17(3): 361-373.

## 4. Saatchi SS, Harris NL, Brown S, Lefsky M, Mitchard ETA, Salas W, et al. Benchmark map of forest carbon stocks in tropical regions across three continents. Proceedings of the National Academy of Sciences 2011, 108(24): 9899-9904.

## 5. Dubayah R, Blair JB, Goetz S, Fatoyinbo L, Hansen M, Healey S, et al. The Global Ecosystem Dynamics Investigation: High-resolution laser ranging of the Earth’s forests and topography. Science of remote sensing 2020, 1: 100002.

## 6. Réjou-Méchain M, Muller-Landau HC, Detto M, Thomas SC, Toan T, Saatchi SS. Local spatial structure of forest biomass and its consequences for remote sensing of carbon stocks. Biogeosciences 2014, 11.

## 7. Labriere N, Tao S, Chave J, Scipal K, Le Toan T, Abernethy K, et al. In situ reference datasets from the TropiSAR and AfriSAR campaigns in support of upcoming spaceborne biomass missions. IEEE Journal of Selected Topics in Applied Earth Observations and Remote Sensing 2018, 11(10): 3617-3627.

## 8. Esteban J, McRoberts RE, Fernández-Landa A, Tomé JL, Marchamalo M. A model-based volume estimator that accounts for both land cover misclassification and model prediction uncertainty. Remote Sensing 2020, 12(20): 3360.

## 9. IPCC. 2006 IPCC Guidelines for National Greenhouse Gas Inventories, Prepared by the National Greenhouse Gas Inventories Programme: IGES, Japan; 2006.

## 10. Baccini A, Goetz SJ, Walker WS, Laporte NT, Sun M, Sulla-Menashe D, et al. Estimated carbon dioxide emissions from tropical deforestation improved by carbon-density maps. Nature Clim Change 2012, 2(3): 182-185.

## 11. Avitabile V, Herold M, Heuvelink GB, Lewis SL, Phillips OL, Asner GP, et al. An integrated pan‐tropical biomass map using multiple reference datasets. Global change biology 2016, 22(4): 1406-1420.

## 12. Bouvet A, Mermoz S, Le Toan T, Villard L, Mathieu R, Naidoo L, et al. An above-ground biomass map of African savannahs and woodlands at 25m resolution derived from ALOS PALSAR. Remote Sensing of Environment 2018, 206: 156-173.

## 13. Ploton P, Mortier F, Réjou-Méchain M, Barbier N, Picard N, Rossi V, et al. Spatial validation reveals poor predictive performance of large-scale ecological mapping models. Nature communications 2020, 11(1): 1-11.

## 14. Schepaschenko D, Chave J, Phillips OL, Lewis SL, Davies SJ, Réjou-Méchain M, et al. The Forest Observation System, building a global reference dataset for remote sensing of forest biomass. Scientific Data 2019, 6(1): 198.

## 15. Carreiras JMB, Vasconcelos MJ, Lucas RM. Understanding the relationship between aboveground biomass and ALOS PALSAR data in the forests of Guinea-Bissau (West Africa). Remote Sensing of Environment 2012, 121(0): 426-442.

## 16. KFS. Field Manual for Biophysical Forest Resources Assessment in Kenya. Improving Capacity in Forest Resources Assessment in Kenya (IC-FRA). Kenya Forest Service. Nairobi; 2016.

## 17. Carreiras J, Melo J, Vasconcelos M. Estimating the Above-Ground Biomass in Miombo Savanna Woodlands (Mozambique, East Africa) Using L-Band Synthetic Aperture Radar Data. Remote Sensing 2013, 5(4): 1524-1548.

## 18. Nwobi CJ, Williams M. Natural and Anthropogenic Variation of Stand Structure and Aboveground Biomass in Niger Delta Mangrove Forests. Frontiers in Forests and Global Change 2021, 4.

## 19. FAO. Global forest resources assessment 2010: Food and Agriculture Organization of the United Nations; 2010 2010.

20. Hansen MC, Potapov PV, Moore R, Hancher M, Turubanova SA, Tyukavina A, et al. High-Resolution Global Maps of 21st-Century Forest Cover Change. Science 2013, 342(6160): 850-853.
